# Gut Microbiota Modulates Obesity‐Associated Skeletal Deterioration Through Macrophage Aging and Grancalcin Secretion

**DOI:** 10.1002/advs.202502634

**Published:** 2025-05-11

**Authors:** Min Huang, Mei Huang, Ling Liu, Fang Yang, Chen He, Yu‐Chen Sun, Yu‐Rui Jiao, Xiang Tang, Jing Hou, Kai‐Xuan Chen, Wen‐Zhen He, Jie Wei, Hui‐Ling Chen, Xia Li, Chao Zeng, Guang‐Hua Lei, Chang‐Jun Li

**Affiliations:** ^1^ Department of Endocrinology Endocrinology Research Center Xiangya Hospital Central South University Changsha 410008 China; ^2^ Department of General Medicine The Fifth Affiliated Hospital of Xinjiang Medical University Urumqi 830000 China; ^3^ Department of Clinical Laboratory Xiangya Hospital Central South University Changsha 410008 China; ^4^ Hunan Key Laboratory of Joint Degeneration and Injury Changsha 410008 China; ^5^ Department of Orthopaedics Xiangya Hospital Central South University Changsha 410008 China; ^6^ Department of Epidemiology and Health Statistics Xiangya School of Public Health Central South University Changsha 410008 China; ^7^ Key Laboratory of Aging‐related Bone and Joint Diseases Prevention and Treatment Ministry of Education Xiangya Hospital Central South University Changsha 410008 China; ^8^ Department of Endocrine Subspecialty of Gerontology Xiangya Hospital, Central South University Changsha 410008 China; ^9^ National Clinical Research Center for Geriatric Disorders Xiangya Hospital Central South University Changsha 410008 China; ^10^ FuRong Laboratory Changsha 410008 China; ^11^ Laboratory Animal Center Xiangya Hospital Central South University Changsha 410008 China

**Keywords:** obesity, skeletal deterioration, immunosenescence, grancalcin, gut‐microbiota

## Abstract

Obesity is associated with skeletal deterioration and increased fracture risk, but the underlying mechanism is unclear. Herein, it is shown that obese gut microbiota promotes skeletal deterioration by inducing bone marrow macrophages (BMMs) senescence and grancalcin (GCA) secretion. Obese mice and those receiving obese fecal microbiota transplants exhibit increased senescent macrophages and elevated GCA expression in the bone marrow. In a study of 40 participants, it is found that obese patients are associated with higher serum GCA levels. It is further revealed that obese gut‐microbiota derived lipopolysaccharides (LPS) stimulate GCA expression in senescent BMMs via activating Toll‐like receptor 4 pathway. Mice with depletion of the *Gca* gene are resistant to the negative effects of obesity and LPS on bone. Moreover, neutralizing antibody against GCA mitigates skeletal deterioration in obese mice and LPS‐induced chronic inflammation mouse model. The data suggest that the interaction between gut microbiota and the immune system contributes to obesity‐associated skeletal deterioration, and targeting senescent macrophages and GCA shows potential of protecting skeletal health in obese population.

## Graphical abstract

Obese gut‐microbiota derived lipopolysaccharides (LPS) induce bone marrow macrophage senescence and GCA secretion via activating TLR4/NF‐κB/MAPKs pathway. Clinically, obese patients are associated with higher serum GCA levels. Mice with depletion of GCA gene show resistance to skeletal deterioration caused by obesity and LPS‐induced chronic inflammation. GCA‐neutralizing antibody shows potential of ameliorating skeletal deterioration in obese and chronic inflammation mouse model.

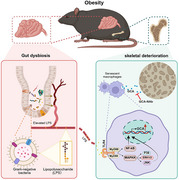

## 1 Introduction

Obesity is a major public health concern, increasing the risk of chronic diseases and fractures.^[^ ^1^, ^2^, ^3^ ^]^ Obesity robustly impairs skeletal microstructure and bone homeostasis, leading to a higher risk of bone fractures despite increased and/or unchanged bone mineral density (BMD).^[^ ^4^, ^5^, ^6^, ^7^ ^]^ Our research and others have confirmed that obese mice exhibit decreased bone mass and increased bone marrow fat, contributing to skeletal deterioration.^[^ ^4^, ^7^, ^8^ ^]^ However, the underlying mechanism remains unclear. Understanding these mechanisms is crucial for developing strategies to protect bone health in obesity.

Obesity disrupts gut microbiota composition, termed “gut dysbiosis”.^[^ ^9^, ^10^ ^]^ Obesity‐associated microbiota alters host energy harvesting, inflammation and insulin resistance,^[^ ^11^, ^12^, ^13^ ^]^ suggesting that gut microbiota is a vital contributing factor to the pathophysiology of obesity. Thus, gut dysbiosis may play a role in deteriorating obesity‐related tissues. Obesity is associated with chronic low‐grade inflammation.^[^ ^14^, ^15^ ^]^ The interaction between gut microbiota and the immune system plays a central role in modulating the immune system and tissue homeostasis throughout life.^[^ ^16^, ^17^, ^18^, ^19^ ^]^ Therefore, we speculate that obese microbiota may alter immune system function and induce obesity‐associated skeletal deterioration.

Recent studies highlight obesity‐associated immunosenescence, where senescent macrophages drive obesity‐induced inflammation and disorder of lipid metabolism.^[^ ^20^, ^21^, ^22^ ^]^ Our previous work showed that macrophages in obese mice contribute to skeletal deterioration via paracrine signaling.^[^ ^4^ ^]^ In addition, we reported that senescent macrophages secret grancalcin (GCA), promoting bone loss and impairing fracture healing in aging mice.^[^ ^23^, ^24^ ^]^ However, several questions remain unanswered: What is the role of obese gut microbiota in immunosenescence? How do gut microbiota‐immune cell interactions regulate macrophage senescence and secretion of GCA? What are the effects of GCA on obesity‐associated skeletal deterioration?

Here, we demonstrate that obese gut microbiota induces bone marrow macrophages (BMMs) senescence and GCA secretion. Obesity is associated with elevated GCA levels in both mice and humans. Mice with knockout of *Gca* gene are resistant to obesity‐induced skeletal deterioration. We further revealed that obese microbiotas‐derived lipopolysaccharides (LPS) promote GCA secretion in BMMs via activation of the Toll‐like receptor 4 (TLR4) pathway. Depletion of the *Gca* gene abolished the negative effects of LPS on bone. Moreover, we identified a neutralizing antibody against GCA, which showed a strong ability to protect skeletal health in obese and LPS‐induced chronic inflammation mouse models.

## 2 Results

### 2.1 Senescent Bone Marrow Macrophages Induce Skeletal Deterioration in Obese Mice via GCA Secretion

**Figure 1:**
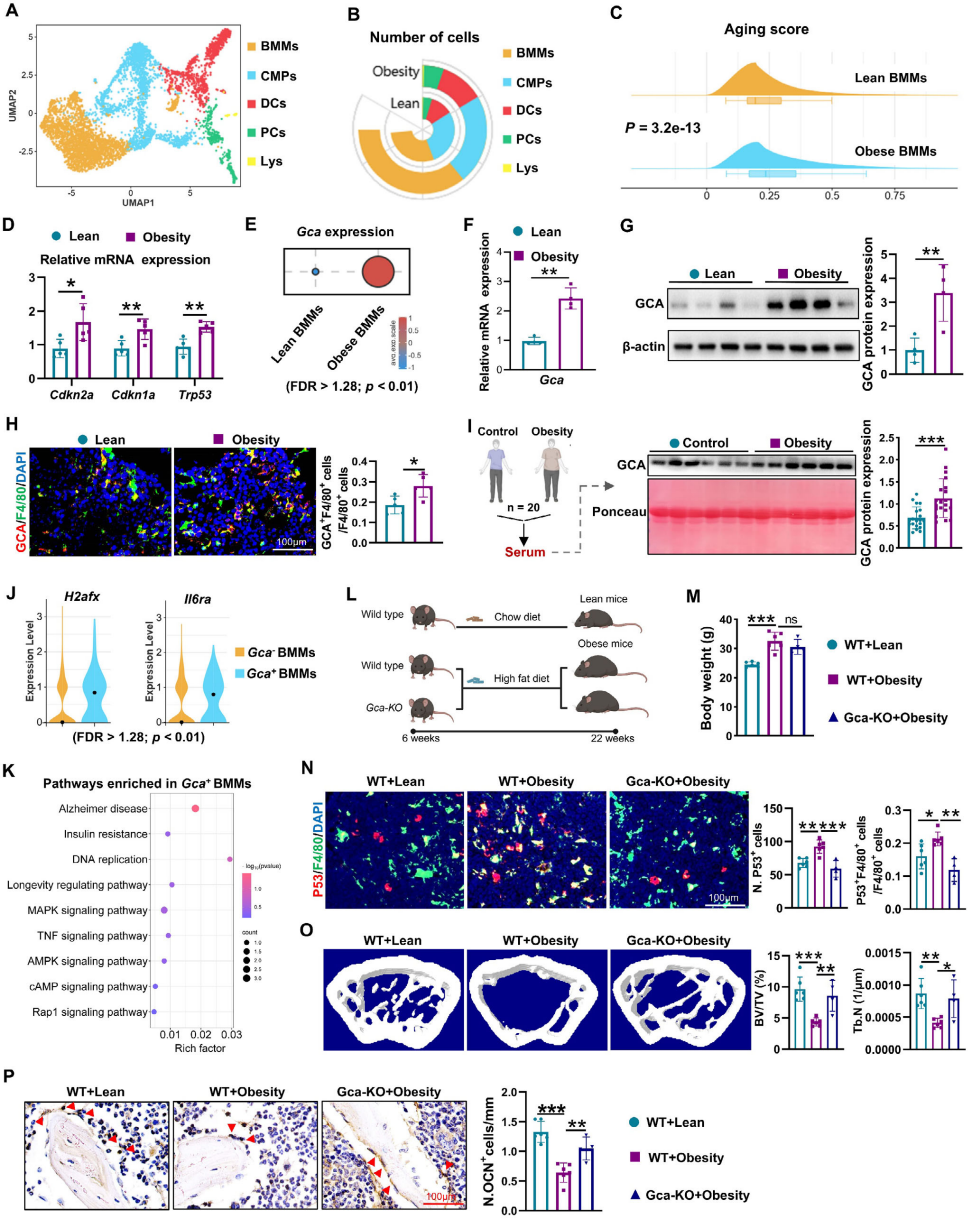
Senescent bone marrow macrophages induce skeletal deterioration in obese mice via GCA secretion. A) UMAP plot showing the main cell types of scRNA‐seq of non‐lymphoid myeloid leucocytes from the bone marrow of obese mice (GSE131834). BMMs, Bone marrow macrophages; CMPs, Common monocyte progenitors; DCs, Dendritic cells; PCs, Progenitor cells; Lys, Lymphocytes. B) Circular stacked bar plot showing the number of cells in lean group and obese group. C) Ridge plot demonstrating that obese BMMs have higher expression of gene sets for senescence. D) qPCR was used to detect the relative expression of the *Cdkn2a, Cdkn1a* and *Trp53* genes in the BMMs of the lean and obese mice (n = 5). E) Bubble diagram showing the expression of *Gca* gene in BMMs of the lean and obese mice. F,G) The relative expression level of GCA in BMMs of lean and obese mice was determined by qPCR and western blot (n = 4). H) Representative double‐immunofluorescent staining images and quantitative analysis of GCA (red) and F4/80 (green) in femurs from lean and obese mice. Nucleuses were stained with DAPI. Scale bar = 100 µm, n = 5. I) Serum was collected from patients with obesity or health controls, and the serum GCA concentrations were tested by western blot (n = 20). Ponceau served as control. J) Violin plots showing that *Gca*‐positive BMMs positively correlate with senescence‐related genes in obese mice. K) KEGG analysis showing the enriched pathways for the differentially expressed genes in *Gca‐*positive BMMs vs *Gca‐*negative BMMs. L) 6 weeks old male *Gca* knockout mice (*Gca‐KO*) and littermate control mice (WT) were fed with normal diet or high‐fat diet for 4 months (n = 4–6). Created with BioRender. M) The body weight of mice. N) Representative images of the femur immunofluorescence staining, immunostained with P53 (red) and F4/80 (green) antibodies (scale bar = 100 µm). Nucleuses were stained with DAPI. O) Representative images showing trabecular architecture by micro‐CT reconstruction in the distal femurs and quantitative analysis of the bone morphological parameters. P) Representative images of osteocalcin (OCN) staining and quantification of the number of osteoblasts (scale bar = 100 µm). Data are shown as the mean ± SD. *P < 0.05; **P < 0.01; ***P < 0.001 according to two‐tailed Student's *t* test (except (M‐P) by one‐way ANOVA).

To assess the impact of obesity on bone metabolism, male mice were fed a high‐fat diet (60% kcal energy as fat) for 16 weeks. In contrast to lean mice, obese mice exhibited increased senescent cells in bone marrow, reduced osteoblasts, and lower bone mass (Figure S1, Supporting Information). Next, we wondered which type of cells initiate these skeletal changes in the context of obesity. Our and others studies reported that BMMs drive multiple aging‐related dysfunctions and participate in the occurrence of obesity associated metabolic disorders.^[^ ^4^, ^23^, ^24^, ^25^ ^]^ Therefore, we speculate that BMMs in obese bone marrow may induce skeletal deterioration. To test this speculation, we performed a bioinformatics analysis using a single‐cell RNA sequencing (scRNA‐seq) dataset of bone marrow cells from lean and obese mice. We found that BMMs became senescent in obesity, as evidenced by the increased aging score and higher expression of senescence‐specific genes, including *Cdkn2a*, *Cdkn1a* and *Trp53*, in obese BMMs compared with lean controls (**Figure** **1**A–D). Our previous study reported that senescent macrophages secrete GCA leading to bone loss during aging.^[^ ^23^ ^]^ Thus, we tested whether senescent BMMs derived GCA could promote obese‐related bone deterioration. We discovered that the expression level of *Gca* was increased in the BMMs of obese mice compared with in lean mice using scRNA‐seq analysis (Figure 1E). Higher levels of GCA in obese BMMs were confirmed by qPCR, western blotting, and immunofluorescence staining (Figure 1F–H). Moreover, in a study of 40 participants (20 obese and 20 healthy controls) (Table S1, Supporting Information), we found that patients with obesity were associated with higher serum GCA levels (Figure 1I). To further explore the association between GCA and senescence in obesity, we divided BMMs into *Gca*‐positive and *Gca*‐negative BMMs. We found that senescence‐related genes were upregulated and senescence‐related diseases and pathways were enriched in *Gca*‐positive BMMs (Figure 1J,K).

The positive correlation between GCA expression in BMMs and skeletal deterioration in obese mice prompted us to investigate the effect of GCA on obesity‐related skeletal disorders. *Gca* gene knockout mice (*Gca‐KO*) were generated and fed a high‐fat diet or normal chow diet for 16 weeks (Figure 1L). We performed immunofluorescence staining to demonstrate that the fluorescence intensity of GCA was dramatically decreased in obese *Gca‐KO* mice compared with obese control mice (Figure S2, Supporting Information). Wild‐type obese mice displayed an increased number of P53^+^ senescent cells in the bone marrow (especially senescent BMMs), low bone mass, and decreased osteoblasts on the bone surface compared with lean controls, whereas *Gca‐KO* mice showed resistance to the obesity‐induced phenotypes of skeletal deterioration (Figure 1M–P). These findings suggest that senescent BMM‐derived GCA contributes to skeletal deterioration in obese mice.

### 2.2 Obese Gut Microbiota Drives Skeletal Deterioration via GCA Secretion from Macrophages

**Figure 2:**
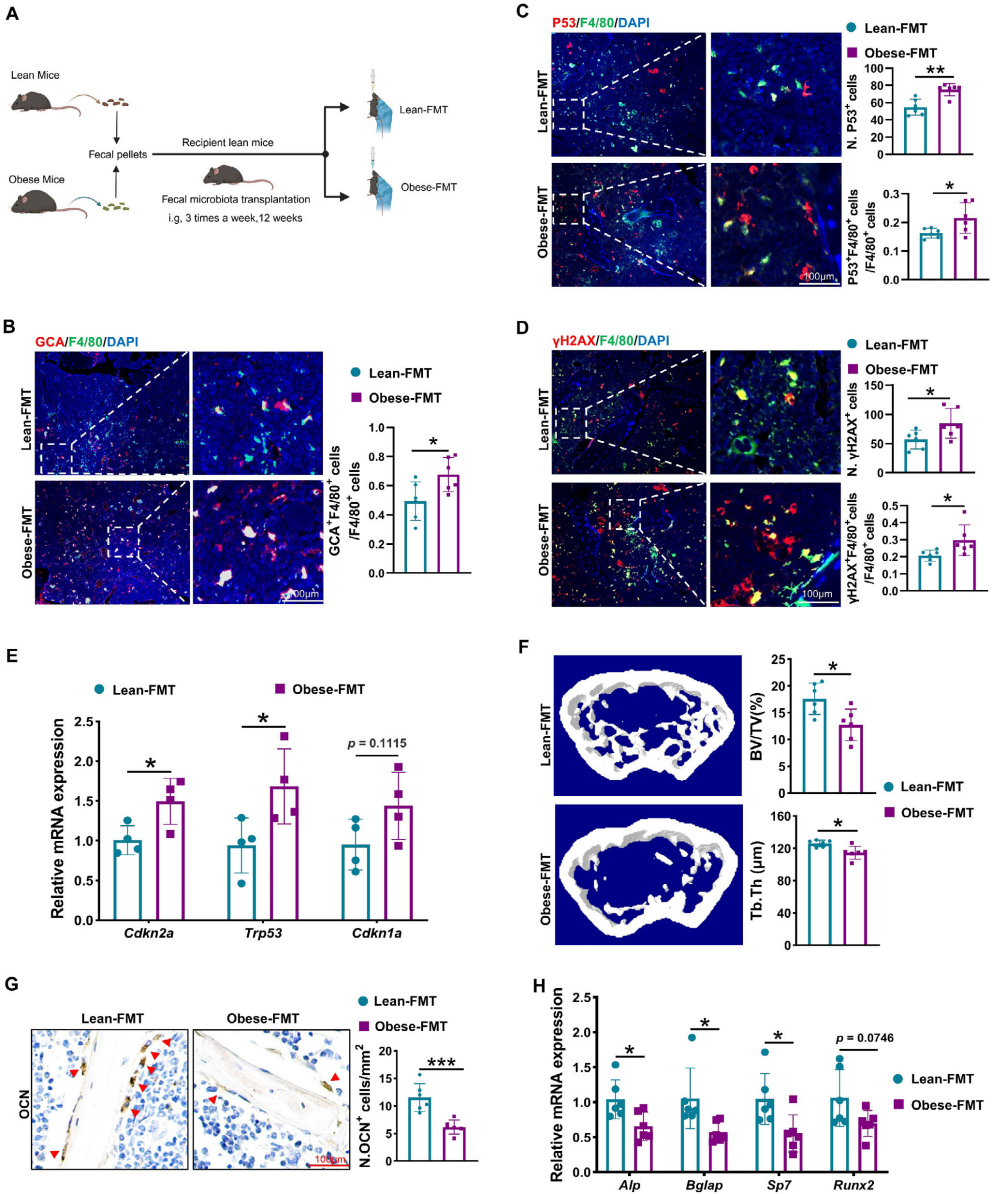
Obese gut microbiota drives skeletal deterioration via GCA secretion from macrophages. A) Schematic illustration of the fecal microbial transplantation (FMT) experiment. Created with BioRender. B) Representative double‐immunofluorescent staining images and quantitative analysis of GCA (red) and F4/80 (green) in femurs of mice receiving fecal from lean mice or obese mice. Nucleuses were stained with DAPI. Scale bar = 100 µm, n = 6. C,D) Representative images of the femur immunofluorescence staining for F4/80, P53 and γH2AX. The number of F4/80^+^ P53^+^ cells and F4/80^+^ γH2AX^+^ cells were quantified (scale bar = 100 µm, n = 6). Nucleuses were stained with DAPI. E) qPCR was used to detect the relative expression of *Cdkn2a*, *Trp53* and *Cdkn1a* genes in the tibial tissue of the lean‐FMT and obesity‐FMT mice (n = 4). F) Representative micro‐CT images of femur and quantitative analysis of trabecular bone volume (BV/TV) and trabecular bone thickness (Tb.Th) (n = 6). G) Representative images of OCN staining of the femur and quantitative analysis of the number of osteoblasts (scale bar = 100 µm, n = 6). H) qPCR was used to detect the relative expression of the osteogenesis‐related genes in the tibial tissue of the lean‐FMT and obesity‐FMT mice (n = 6). Data are shown as the mean ± SD. *P < 0.05; **P < 0.01; ***P < 0.001; Student's *t* test.

Next, we wondered how obesity induce BMMs senescence and GCA secretion, thus leading to skeletal deterioration. The gut microbiota is involved in a variety of obesity‐related metabolic disorders.^[^ ^10^, ^26^ ^]^ We explored whether gut microbiota could regulate obesity‐related skeletal deterioration by influencing GCA secretion from BMMs. We transplanted the fecal microbiota from obese mice into lean recipient mice (obese‐FMT mice) three times a week for 12 weeks, with lean recipient mice transplanted with lean fecal microbiota as a control (lean‐FMT mice) (**Figure** **2**A). After fecal microbiota transplantation (FMT), the GCA levels in BMMs were higher in obese‐FMT mice than in lean‐FMT mice (Figure 2B). The obese FMT mice displayed elevated senescent cells in femurs, including senescent BMMs, as evidenced by increased fluorescence intensity of P53 and γ‐H2AX (Figure 2C,D). In addition, senescence‐related genes, including *Cdkn2a* and *Trp53*, were upregulated in the bone tissue of obese‐FMT mice compared with those in lean‐FMT mice (Figure 2E). Subsequently, microcomputed tomography (Micro‐CT) analysis showed that trabecular bone mass was lower in obese‐FMT mice than in lean‐FMT control mice (Figure 2F). Osteoblasts on the trabecular bone surface and expression levels of osteoblast‐specific genes were significantly lower in obese‐FMT mice than in control mice (Figure 2G,H). In addition, no significant differences were observed in the body weights of recipient mice with lean or obese FMT (Figure S3, Supporting Information). These above data indicate that microbiota transplantation from obese mice leads to GCA secretion from BMMs and skeletal deterioration without affecting mechanical loading.

### 2.3 Obese Gut Microbiota‐Derived LPS Stimulates GCA Secretion

**Figure 3:**
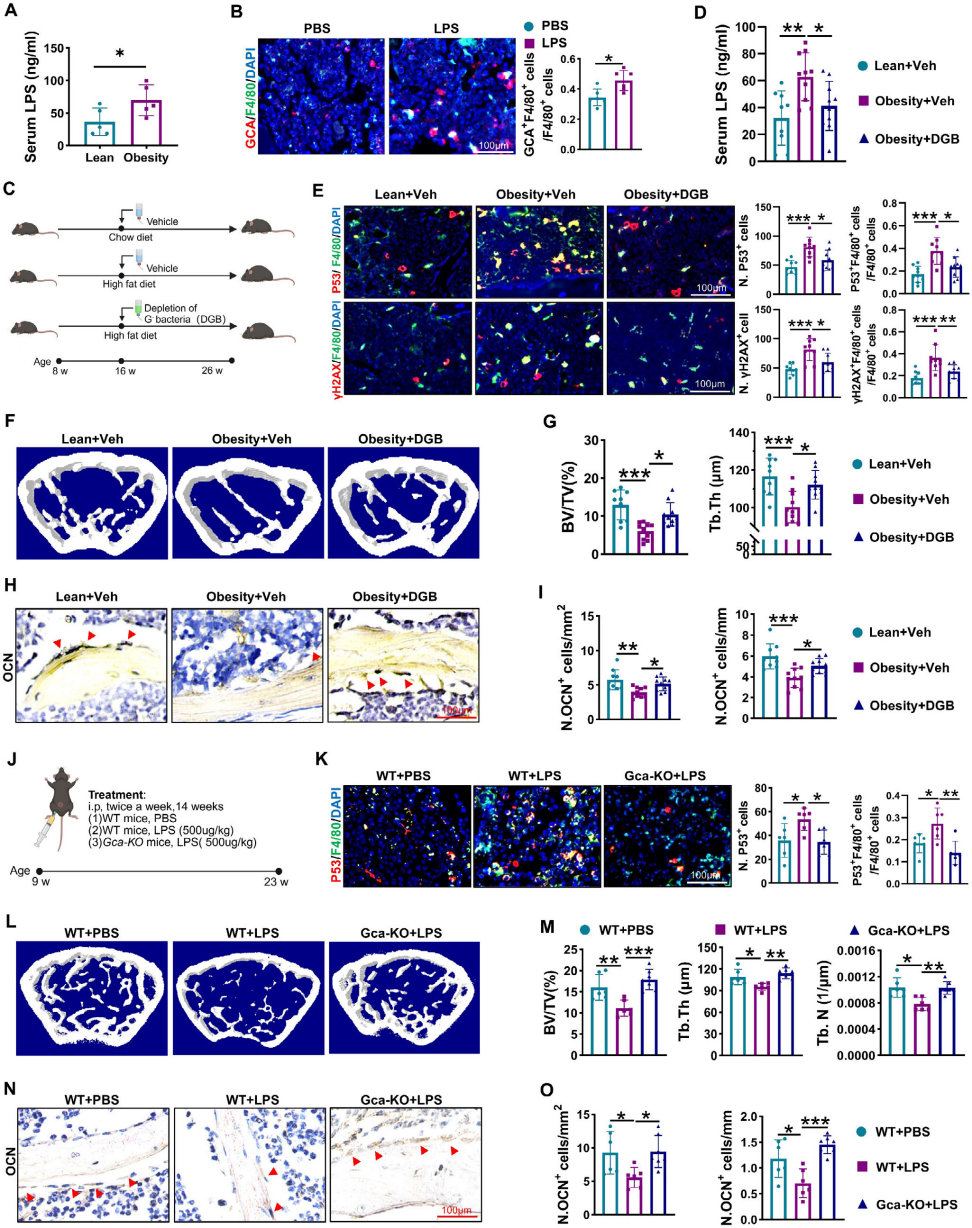
Obese gut microbiota‐derived LPS stimulates GCA secretion. A) Serum LPS levels were measured by ELISA kits from lean or obese mice (n = 5). B) 9 weeks old male wild type mice received intraperitoneal injections of phosphate buffer saline (PBS) or LPS for 14 weeks. Representative images of femur immunofluorescence staining for GCA (red), F4/80(green) and DAPI (blue), with quantitative analysis of double positive cells. Scale bar = 100 µm, n = 6. C) Schematic diagram illustrating the application of antibiotic water to eradicate gram‐negative bacteria in obese mice (n = 9–10). Created with BioRender. D) Serum LPS concentrations were measured by ELISA kits. E) Representative images of the femur immunofluorescence staining for F4/80, P53 and γH2AX. The number of F4/80^+^ P53^+^ cells and F4/80^+^ γH2AX^+^ cells were quantified (scale bar = 100 µm). Nucleuses were stained with DAPI. F,G) Representative micro‐CT images of femur and quantitative analysis of trabecular bone volume (BV/TV) and trabecular bone thickness (Tb.Th). H,I) Representative images of OCN staining of the femur and quantitative analysis of the number of osteoblasts (scale bar = 100 µm). J) 9 weeks old male *Gca‐KO* and littermate control mice (WT) treated with LPS or vehicle by intraperitoneal injection (n = 6–7). Created with BioRender. K) Representative images of femur immunofluorescence staining of P53 (red), F4/80 (green) and DAPI (blue) and the number of P53 and F4/80 double positive cells was quantified (scale bar = 100 µm). L,M) Representative micro‐CT images of femur and quantitative analysis of trabecular bone volume, thickness and number. N,O) Representative images of OCN staining and quantitative analysis of the OCN‐positive cells (scale bar = 100 µm). Data are shown as the mean ± SD. *P < 0.05; **P < 0.01; ***P < 0.001 according to one‐way ANOVA (except (A and B) by Student's *t* test).

Next, we tested how obese gut microbiota regulate BMMs senescence and GCA secretion. Previous studies show that obesity alters gut microbiota composition with increased LPS‐bearing gram‐negative bacteria, which triggers the metabolic endotoxemia in obesity.^[^ ^27^, ^28^, ^29^, ^30^ ^]^ Next, we wondered whether obese gut microbiota‐derived LPS promotes GCA secretion from macrophages and skeletal deterioration. First, we confirmed that serum LPS concentrations were higher in obese mice than in lean controls (**Figure** **3**A). Then, we constructed a chronic low‐inflammatory mouse model using low‐dose and long‐term LPS administration.^[^ ^29^, ^31^ ^]^ Immunofluorescence staining revealed that LPS‐treated mice had higher GCA levels in macrophages than in vehicle‐treated mice (Figure 3B). Moreover, decreased bone mass and osteoblasts, and increased senescent cells in the bone marrow were observed in LPS treatment mice compared with controls (Figure S4, Supporting Information). To further investigate whether the destructive effects of obesity on bone were dependent on the presence of gut microbiota‐derived LPS, we treated obese mice with antibiotics against gram‐negative bacteria (Figure 3C). To confirm the efficiency of the antibiotics, we conducted an Enzyme‐Linked ImmunoSorbent Assay (ELISA) test to detect LPS levels, and the data showed that serum LPS concentrations decreased in obese mice supplied with antibiotics against gram‐negative bacteria compared with vehicle‐treated obese mice (Figure 3D). Consistent with the above results, obese mice showed increased P53^+^ and γH2AX^+^ senescent cells and decreased trabecular bone volume, thickness, and osteoblasts (Figure 3E–I). However, the ablation of gram‐negative bacteria by antibiotics almost abolished the negative effects of obesity on the bone (Figure 3E–I). Next, we used GCA‐deficient mice to determine whether GCA mediated the effects of LPS on skeletal deterioration (Figure 3J). The elevated senescent cells, low bone mass, and reduced osteoblasts in the LPS‐treated group were alleviated in *Gca‐KO* mice (Figure 3K–O). These findings indicate that obese gut‐derived LPS induces skeletal deterioration in a GCA‐dependent manner.

### 2.4 LPS Enhances GCA Expression in Macrophages Through Activation of TLR4 Pathway

**Figure 4:**
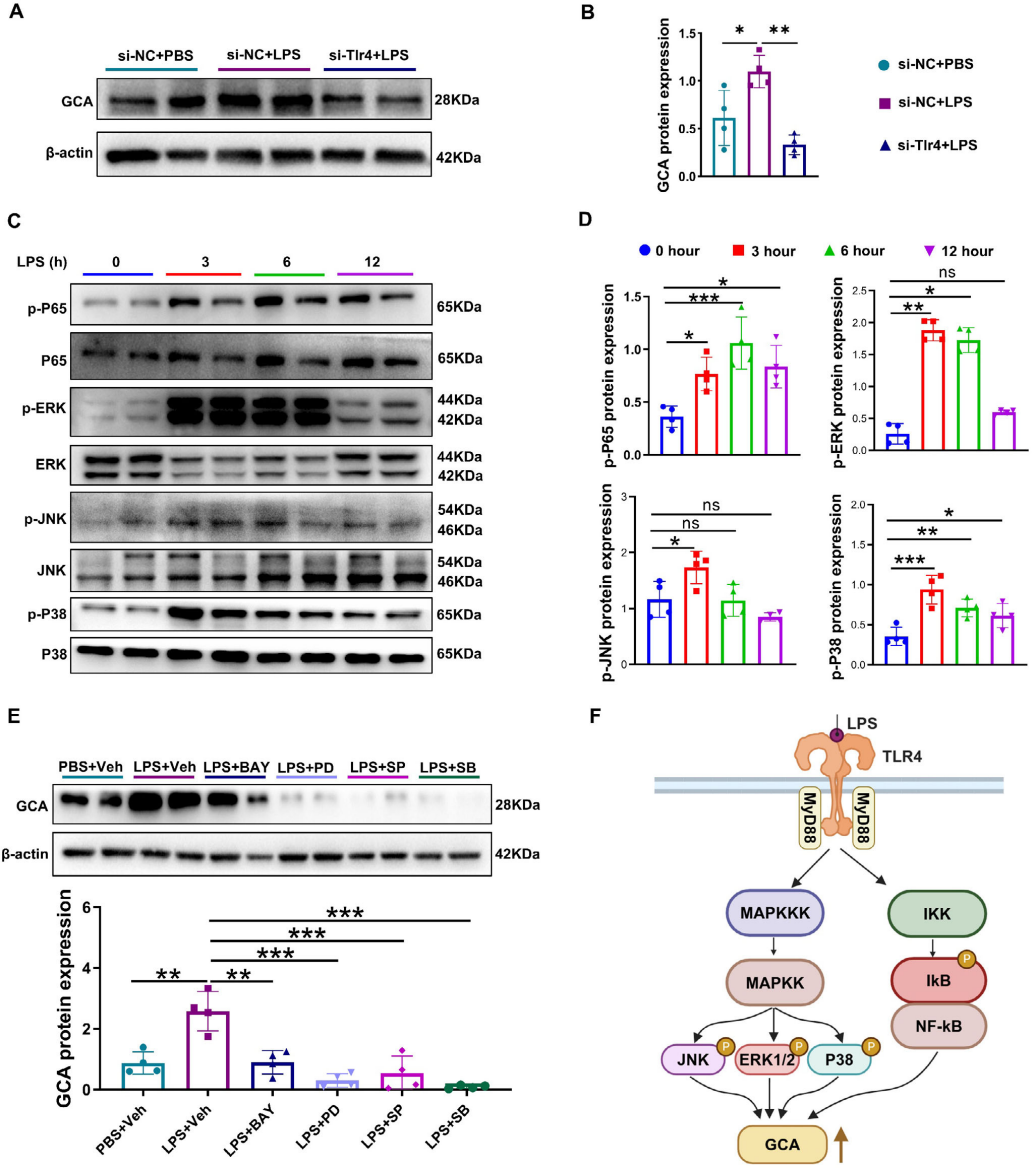
LPS enhances GCA expression in macrophages through activation of TLR4 pathway. A,B) The protein levels of GCA in siRNA‐*Tlr4* or siRNA‐NC treated BMMs supplied with or without LPS for 3 h (n = 4). C,D) Western blot was used to determine these protein expression levels in BMMs treated with LPS at the indicated time points (n = 4). E) BMMs were treated with LPS for 3 h, and these signaling pathway inhibitors were added 1 h before LPS. The expression of GCA was determined by western blot analysis (n = 4). F) Schematic diagram of LPS promoting GCA expression through TLR4‐mediated downstream NF‐κB and MAPKs signaling pathway. Created with BioRender. Data are shown as the mean ± SD. *P < 0.05; **P < 0.01; ***P < 0.001; one‐way ANOVA.

We investigated how LPS promotes GCA expression in BMMs. TLR4 is a critical receptor for LPS transmembrane signal transduction.^[^ ^32^, ^33^ ^]^ Therefore, we **hypothesized that LPS regulates GCA** expression **by activating the TLR4 receptor in BMMs. BMMs were treated with LPS, with additional transfection of** *Tlr4*‐siRNA or control siRNA. We found that LPS enhanced GCA levels in BMMs, which was negated by *Tlr4* knockdown (**Figure** **4**A,B). Next, we wondered whether the NF‐κB and MAPKs (including ERK, JNK and P38) signaling pathway, downstream of the LPS‐TLR4 axis,^[^ ^32^ ^]^ play a role in LPS/TLR4‐induced GCA expression. We confirmed that LPS stimulated the activation of P65, ERK, JNK and P38 in BMMs (Figure 4C,D). Administration of a specific inhibitor of NF‐κB (BAY 11–7082), ERK (PD98059), JNK (SP600125) or P38 (SB203580) blunted or abolished the positive effect of LPS on GCA level in BMMs (Figure 4E). Collectively, these data indicate that LPS induces GCA expression in BMMs through TLR4/NF‐κB/MAPKs signaling pathway (Figure 4F).

### 2.5 GCA‐Neutralizing Antibody Ameliorates Skeletal Deterioration in Obese Mice

**Figure 5:**
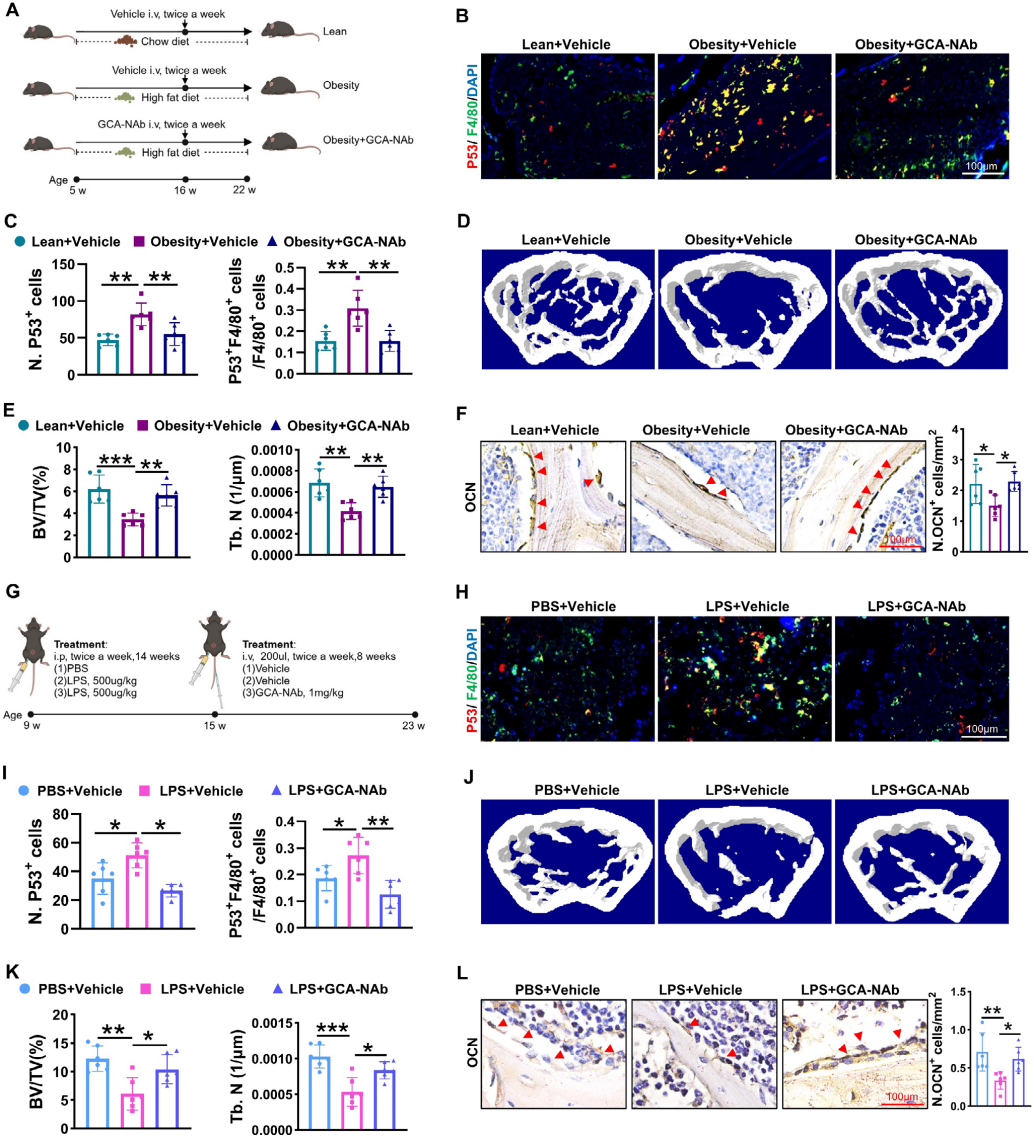
GCA‐neutralizing antibody ameliorates skeletal deterioration in obese mice. A) Schematic diagram of obese mice treated with GCA‐neutralizing antibody (GCA‐NAb) or vehicle (n = 6). Created with BioRender. B,C) Representative images of immunofluorescence staining of P53 (red), F4/80 (green) and DAPI (blue), and the number of P53 and F4/80 double positive cells was quantified (scale bar = 100 µm). D,E) Representative micro‐CT images of femur and quantitative analysis of trabecular bone volume and number. F) Representative images of OCN staining and quantitative analysis of the OCN‐positive cells (scale bar = 100 µm). G) Schematic diagram of the effects of GCA‐Nab on LPS‐induced skeletal deterioration (n = 6). Created with BioRender. H,I) Representative images of immunofluorescence staining of P53 (red), F4/80 (green) and DAPI (blue), and the number of P53 and F4/80 double positive cells was quantified (scale bar = 100 µm). J,K) Representative micro‐CT images and quantitative analysis of trabecular bone volume and number. L) Representative images of OCN staining of the femur and quantification of the number of osteoblasts (scale bar = 100 µm). Data are shown as the mean ± SD. *P < 0.05; **P < 0.01; ***P < 0.001; one‐way ANOVA.

Given the role of GCA in obesity‐related skeletal deterioration, we designed a neutralizing antibody against GCA (GCA‐NAb).^[^ ^23^ ^]^ We treated obese mice with GCA‐NAb twice weekly for 6 weeks (**Figure** **5**A). Immunofluorescent staining showed that GCA‐NAb treatment decreased the number of p53 foci in the bone of obese mice compared with vehicle‐treated mice (Figure 5B,C). We found that bone mass and osteoblasts were increased in GCA‐NAb‐treated obese mice compared with vehicle‐treated controls (Figure 5D–F). Next, we explored the effect of GCA‐NAb on a low‐dose LPS‐induced chronic low‐grade inflammatory mouse model (LPS mice) as a mimic of obesity (Figure 5G). GCA‐NAb treatment significantly protected the mice from LPS‐induced skeletal deterioration. The fluorescence intensity of P53 was significantly reduced in GCA‐NAb‐treated LPS mice compared with vehicle‐treated control mice (Figure 5H,I). Furthermore, GCA‐NAb‐treated mice showed improved bone mass and increased osteoblasts (Figure 5J–L). In addition, GCA‐Nab had no effect on the body weight of obese mice or LPS‐treated mice (Figure S5, Supporting Information). Overall, these data suggest that GCA‐NAb alleviates skeletal deterioration in obese and chronic low‐grade inflammatory mouse models.

## 3 Discussion

Obesity is associated with skeletal deterioration and increased fracture risk.^[^ ^34^, ^35^ ^]^ However, the underlying mechanism is unclear. Our study suggests that obese gut microbiota drives immunosenescence and skeletal deterioration. Transplantation of fecal microbiota from obese mice induces BMMs senescence and GCA secretion, leading to skeletal deterioration. Depletion of the *Gca* gene abolishes the negative effects of obesity and obese gut microbiota‐derived LPS on the bone. Moreover, neutralizing antibody against GCA shows potential of ameliorating skeletal deterioration in obese mice and LPS induced chronic inflammation mouse model.

Obesity increases fracture risk despite normal or higher BMD, a phenomenon known as the “obesity paradox”.^[^ ^36^, ^37^ ^]^ Impaired skeletal quality and aging induced by obesity may contribute to an increased risk of fractures. Recent studies suggest that obesity induces senescent cells accumulation in adipose tissue and other tissues.^[^ ^22^, ^38^, ^39^, ^40^ ^]^ In aged bone marrow, impaired macrophage efferocytosis enables apoptotic osteoblasts to escape osteoimmune surveillance, ultimately leading to skeletal aging.^[^ ^41^ ^]^ Our previous work demonstrated that senescent BMMs drive skeletal and systemic aging via GCA secretion and induction of paracrine senescence in a naturally aging mouse model.^[^ ^23^, ^24^, ^42^ ^]^ Thus, we speculated that obesity may induce BMMs senescence and GCA secretion, leading to skeletal deterioration. scRNA sequencing and immunostaining analyses revealed a dramatic increase in the number of senescent macrophages and GCA expression in the bone marrow of obese mice. GCA is mainly expressed in myeloid cells, including neutrophils and macrophages.^[^ ^23^ ^]^ In this study, depletion of the *Gca* gene abolished the negative effects of obesity on the bone. However, we do realize that other immune cells, such as T cells, may also become senescent and secrete pro‐aging/inflammatory factors, leading to obesity‐associated skeletal deterioration.

The interaction between gut microbiota and the immune system plays a key role in obesity‐related multiple tissue dysfunctions, such as type 2 diabetes.^[^ ^18^, ^43^, ^44^, ^45^ ^]^ However, the role of gut microbiota‐immune system interactions in obesity‐associated skeletal deterioration is unknown. In this study, we found that obese gut microbiota‐immune cell interactions induced skeletal deterioration. Specifically, LPS‐bearing gram‐negative bacteria promoted BMMs senescence and GCA secretion via activation of the LPS‐TLR4 pathway. Our findings showed that LPS levels were increased in obese mice and humans. In addition, we constructed a chronic low‐grade inflammation mouse model induced by low‐dose LPS and observed immunosenescence and GCA secretion of senescent macrophages, similar to the data obtained from obese mice. This mouse model mimics chronic low‐grade inflammation in obese and naturally aging mice and could be utilized in studies of obesity and aging. LPS directly regulates bone loss by enhancing bone resorption;^[^ ^46^ ^]^ however, our data on the resistance to LPS‐induced bone loss in mice with knockout of the *Gca* gene indicate that GCA, at least partially, mediates the negative effect of LPS on bone.

In summary, our data raise a proof of concept that the interaction between gut microbiota and the immune system contributes to obesity‐associated skeletal deterioration. Targeting senescent macrophages and GCA may represent a promising strategy for preserving skeletal health in obesity.

## 4 Experimental Section

### Clinical Samples

Peripheral blood was collected from patients with obesity (Body Mass Index (BMI) > 28) or healthy controls (18.5 < BMI < 24). All the participants were informed about the study and signed an informed consent form. The clinical study was approved by the Medical Ethics Committee of Xiangya Hospital, Central South University (No.202211246). The baseline characteristics of the participants are summarized in Table S1 (Supporting Information). Participants with autoimmune diseases, polycystic ovary syndrome, thyroid or parathyroid diseases, cushing's syndrome, diabetes, hyperprolactinemia, hematological diseases, malignant tumors, organ failure, and those taking medications (such as glucocorticoids, estrogen, thyroid hormones and bisphosphonates) were excluded.

### Animal Models

To establish the obesity mouse model, 5–8 weeks old male mice were fed an ordinary breeding diet or a high‐fat diet (60% fat content, D12492, Wuhan BIOPIKE Bioscience Co. Ltd., China) for 4 months. The weight of the high‐fat group was 20% higher than that of the control group, which served as a simple criterion for modeling.

For fecal microbiota transplantation, recipient mice were treated with combined antibiotics (ABX) (1 g L^−1^ ampicillin sodium, 0.5 g L^−1^ vancomycin hydrochloride, 1 g L^−1^ neomycin and 0.5 g L^−1^ metronidazole) by gavage for one week to deplete gut microbiota before fecal transplantation, as previously described.^[^ ^47^, ^48^ ^]^ The feces of lean or obese mice were collected and soaked in PBS for 15 min. The mixture was vortexed until it dissolved into a suspension and centrifuged at 1000 rpm for 5 min. The supernatant was collected, and the centrifugation was repeated. The final supernatant was mixed with 40% volume of sterile glycerol to achieve a final concentration of 20%. The mixture was stored at −80 °C until transplantation. Each mouse was orally gavaged with 200 µL of the mixed solution at three times a week for 12 weeks, and then the mice were sacrificed to harvest specimens.

To establish the LPS‐induced metabolic endotoxemia mouse model, 9 weeks old C57BL/6 mice were subjected to intraperitoneal injections of LPS (500 µg kg^−1^, twice a week) or vehicle for a duration of 14 weeks, and then the tissue samples were collected. To explore the effects of LPS‐bearing gram‐negative bacteria on obesity‐associated skeletal deterioration, 8‐week‐old C57BL/6 mice were fed a normal chow or high‐fat diet for 8 weeks. Mice on the high‐fat diet were further divided into two groups: one group received water containing antibiotics against gram‐negative bacteria (1 g L^−1^ neomycin and 1 g L^−1^ colistin) for 10 weeks, and the other group received regular tap water. Mice fed on a normal chow diet were also provided with tap water.

To test the therapeutic potential of GCA‐neutralizing antibody (GCA‐NAb) against obesity‐ and LPS‐induced skeletal deterioration, obese mice received GCA‐Nab (1 mg kg^−1^, twice a week) or vehicle treatment through the tail vein for 6 weeks.^[^ ^23^ ^]^ Mice were treated with intraperitoneal injections of PBS or LPS from 9 to 23 weeks of age and received GCA‐Nab (1 mg kg^−1^, twice weekly) or vehicle at 15 weeks of age.

*Gca*‐knockout mice were generated using CRISPR/Cas9 technology at BIORAY LABORATORIES (China). Exon 3 of GCA was selected as the knockout region. The targeting vector was injected into C57BL/6 mouse eggs. For genotyping, genomic DNA was extracted from the tail tips and PCR analysis was conducted. Healthy mice were used for in‐house mating to generate sufficient mice for the experiments.

Wild‐type C57BL/6 male mice were purchased from Shanghai SLAC Laboratory Animal Co., Ltd. All mice were maintained in a standard, specific pathogen‐free facility of the Laboratory Animal Research Center of Central South University at a controlled temperature (22–24 °C), with a 12 h dark/light cycle (07:00 to 19:00 light on), with standard food (Hunan SJA Laboratory Animal Company, China), water provided ad libitum and environmental enrichments. All animal care protocols and experiments were reviewed and approved by the Animal Care and Use Committee of the Laboratory Animal Research Center of the Xiangya Medical School of Central South University (No. 2024030546).

### Micro‐CT Scanning Analysis

The femur was completely fixed with 4% paraformaldehyde, and bone mass was analyzed using micro‐CT (model: Skyscan 1172).^[^ ^49^ ^]^ Scanning parameters: energy value (voltage 65 kV, current 153 µA), focal axis resolution (15 µm). The original data files obtained by scanning were reconstructed using Skyscan image reconstruction software. Subsequently, the 3D spatial position of the femur was adjusted using 3D model visualization software (DATA) to ensure consistency in the positions of all analyzed specimens. Data from three directions‐sagittal plane, transverse, and coronal were saved in the corresponding folders. The cross‐sectional data were further analyzed using CTAn (data analysis software), and the region of interest, the cancellous bone within 3 mm of the femoral growth plate, was drawn. Appropriate parameters were set, and the same parameters were retained for all femurs for comparison. The ratios of trabecular bone volume to total tissue volume (BV/TV), trabecular number (Tb.N), trabecular thickness (Tb.Th), and trabecular separation (Tb.Sp) were calculated.

### Immunohistochemical Staining

Femora were harvested from the mice after euthanasia and fixed in 4% formalin for 24 h. Bone tissues were decalcified in 10% EDTA (Ethylene Diamine Tetraacetic Acid) for 14 days and embedded in paraffin. Five‐micrometer‐thick, longitudinally oriented bone sections were prepared for further staining. Immunohistochemical staining was performed as previously described.^[^ ^50^ ^]^ Bone sections were incubated with a primary antibody against osteocalcin (M137, 1:500, Takara) overnight at 4 °C. Subsequently, the bone slices were incubated with the appropriate secondary antibodies, followed by counterstaining with hematoxylin. For immunohistochemical analysis, the sample area selected for single‐blind analysis was 1 mm^2^ within the metaphyseal secondary spongiosa.

### Immunofluorescence Staining

Immunofluorescence staining was performed as previously described.^[^ ^51^ ^]^ Bone slices were processed for antigen retrieval by digestion with 0.05% trypsin at 37 °C for 15 min, and then incubated with primary antibodies against P53 (sc‐126, 1:100, Santa Cruz), γH2AX (sc‐517348, 1:100, Santa Cruz), GCA (PA5‐77127, 1:200, Invitrogen) and F4/80 (ab6640, 1:400, Abcam) overnight at 4 °C, followed by incubation with FITC‐ or Cy3‐conjugated secondary antibodies (Jackson ImmunoResearch, 1:200). Nuclei were counterstained with DAPI (Sigma‐Aldrich, USA).

### Cell Culture

BMMs were isolated as previously described.^[^ ^49^ ^]^ Bone marrow cells were flushed out and cultured overnight in a‐MEM medium containing 10% FBS (Fetal bovine serum), 100 U mL^−1^ penicillin and 100 µg mL^−1^ streptomycin (complete medium). Cells in the supernatant were collected and cultured in a medium containing 30 ng mL^−1^ M‐CSF (Proteintech) for 3 days. All cells were grown at 37 °C in a 5% CO_2_ humid atmosphere. BMMs were treated with LPS (100 ng mL^−1^, L2880, Sigma), siRNA‐*Tlr4* (RiboBio, China), BAY 11–7082 (10 µmol L^−1^, Selleck, USA), PD98059 (10 µmol L^−1^, Selleck), SP600125 (5 µmol L^−1^, Selleck), SB203580 (10 µmol L^−1^, Selleck) and their control vehicles with different time. Then the cell lyses were collected for further analysis.

### qRT‒PCR Analysis

TRIzol reagent (Accurate Biology) isolated total RNA from tissues and cell lysates. Reverse transcription was performed using 1µg of total RNA by a reverse transcription kit (Accurate Biology). For relative quantitative qRT‐PCR, amplification reactions were set up in 10 µL reaction volumes using the SYBR Green Premix *Pro Taq* HS qPCR Kit (Accurate Biology). Fold changes over controls were calculated using the relative quantification method of 2^−ΔΔCt^. Primer sequences used for qPCR are summarized in Table S2 (Supporting Information).

### Western Blot Analysis

For western blot analysis, total cell lysates and human serum were separated by SDS‐PAGE and blotted on polyvinylidene fluoride membranes (Millipore). The membranes were incubated with corresponding primary antibody against to GCA (PA5‐77127, 1:1000, Invitrogen), p‐JNK (80024‐1‐RR,1:1000, Proteintech), JNK (80024‐1‐RR66210‐1‐Ig, 1:3000, Proteintech), p‐P38 (9211, 1:1000, CST), P38(9212, 1:1000, CST), p‐P65(3033, 1:1000, CST), P65(8242, 1:1000, CST), p‐ERK (4370, 1:2000, CST), ERK (4695, 1:1000, CST) and β‐actin (BA2305, 1:4000, Boster) at 4 °C overnight, and then reexamined with secondary antibodies labeled with horseradish peroxidase. The blots were detected using enhanced chemiluminescence (ECL Kit; Amersham Biosciences).

### ELISA Analysis

The concentration of LPS was measured using an ELISA kit (MBS261904, MYBIOSOURCE) according to the manufacturer's instructions.

### Bioinformatics Analysis of Single‐Cell RNA‐Seq

The accession number for the single‐cell RNA sequencing data reported in this study is GSE131834. Bioinformatics analysis was performed using Omicsmart, a dynamic, real‐time, interactive online platform for data analysis (http://www.omicsmart.com).

### Statistics Analysis

Data were analyzed and mapped using GraphPad Prism software (version 8.0). Two‐tailed Student's *t*‐test was used to compare the differences between the two groups. One‐way ANOVA analysis was used to compare the differences between multiple groups. All experiments were repeated thrice to guarantee the reproducibility of the findings, and representative experiments were shown. The experimental data were represented by mean ± SD, in which *P* < 0.05 indicated the data were statistically different.

## Conflict of Interest

The authors declare no conflict of interest.

## Author Contributions

M.H. and M.H. contributed equally to this work. C.J.L., M.H., and M.H. designed the experiments. M.H. and M.H. performed the experiments and analyzed the data. L.L., F.Y., Y.C.S., W.Z.H., and X.T. helped to collect samples. Y.R.J., C.H., J.H., X.T., and K.X.C. provided technical assistance. J.W., H.L.C., X.L., C.Z., G.H.L., and C.J.L. supervised the study. M.H. and M.H. drafted the manuscript, and C.J.L. wrote and revised the manuscript.

## Supporting information



Supporting Information

## Data Availability

The data that support the findings of this study are available from the corresponding author upon reasonable request.
