# Supplementary material for: Gut Microbiota Modulates Obesity‐Associated Skeletal Deterioration Through Macrophage Aging and Grancalcin Secretion
Source: Adv Sci (Weinh). 2025 May 11;12(28):2502634. doi: 10.1002/advs.202502634 (PMC12302596; doi:10.1002/advs.202502634)
Supplement: Supplementary file 1 — Supporting Information [file ADVS-12-2502634-s001.docx]

**Supplementary figures**


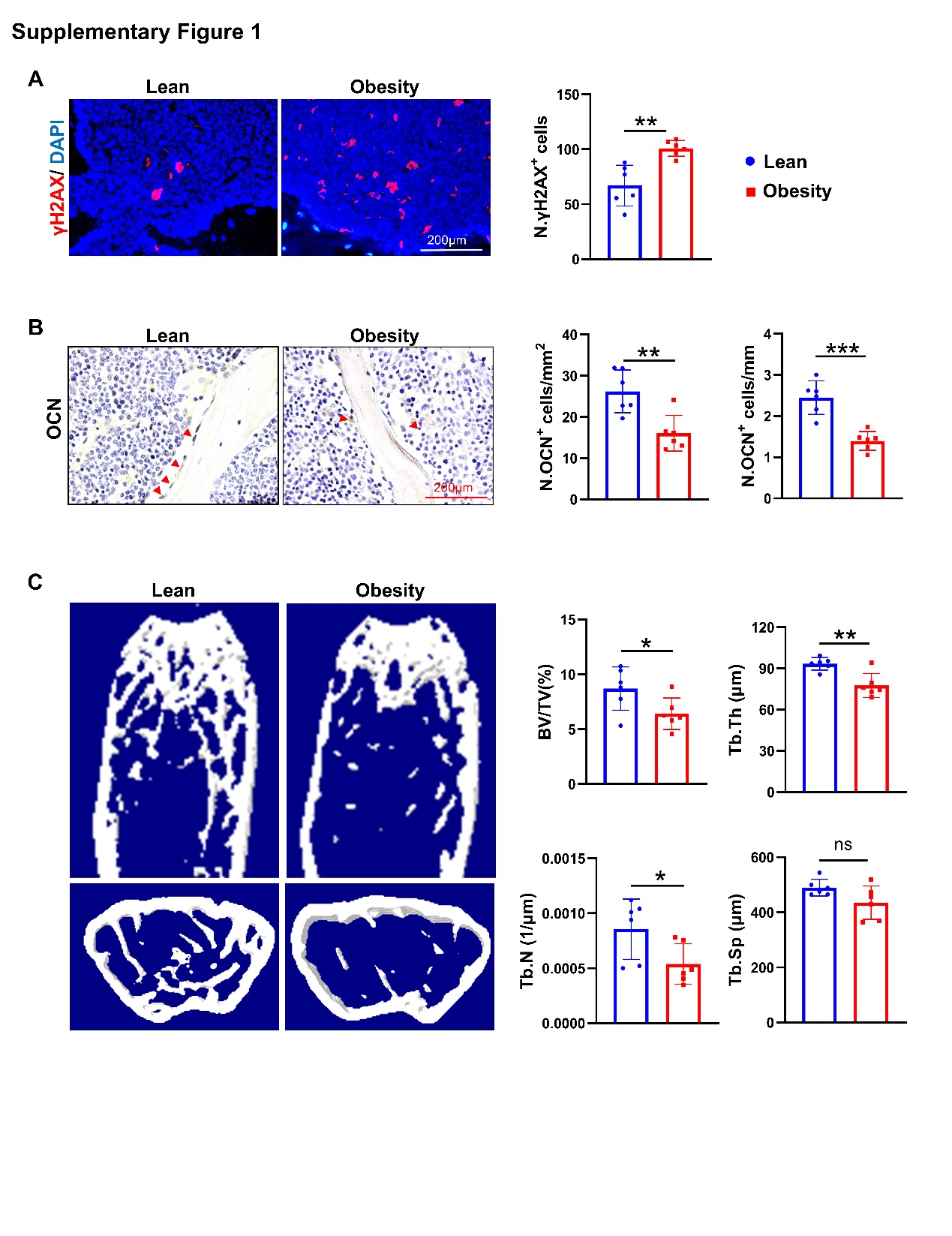


**Figure S1. Obesity leads to skeletal deterioration**

(A) Representative immunofluorescence staining images and quantitative analysis of γH2AX-posotive cells (red) in femur from high-fat diet induced obese mice and control lean mice (scale bar = 200 μm, n = 6).

(B) Representative images of OCN staining of the femur and quantification of the number of OCN-positive osteoblasts (scale bar = 200 μm, n = 6).

(C) Representative micro-CT images of femurs and quantitative analysis of trabecular volume (BV/TV), trabecular thickness (Tb.Th), trabecular number (Tb.N) and trabecular separation (Tb.Sp) (n = 6).

Data are shown as the mean ± SD. **P* <0.05; ***P* <0.01; ****P* <0.001; Student’s *t* test.

**
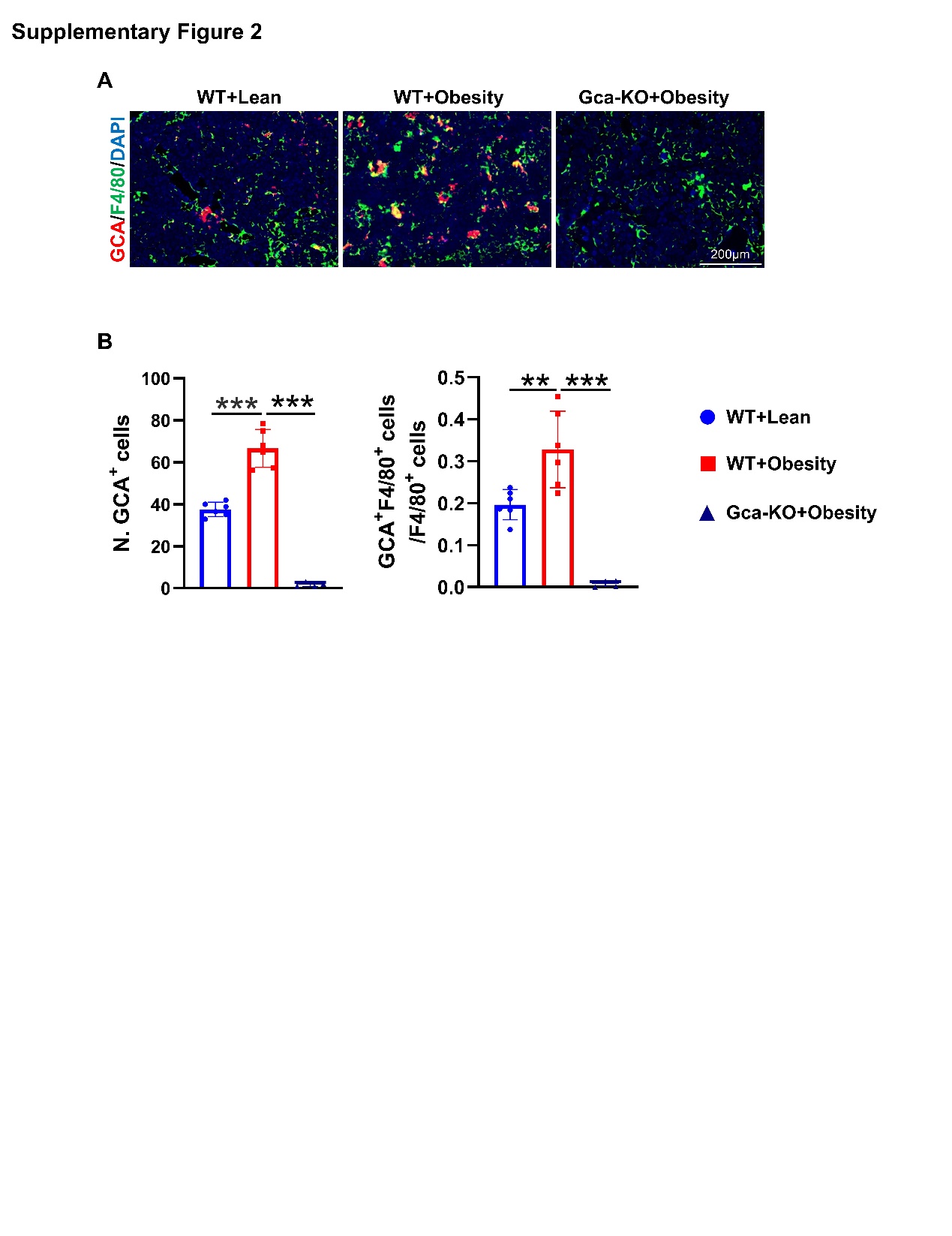
**

**Figure S2. Knockout efficiency of obese *Gca-KO mice***

(A and B) Representative double-immunofluorescent staining images and quantitative analysis of GCA (red) and F4/80 (green) in femurs from lean and obese mice. Nucleuses were stained with DAPI. Scale bar = 200 μm, n = 4-6.

Data are shown as the mean ± SD. **P* <0.05; ***P* <0.01; ****P* <0.001; one-way ANOVA.

**
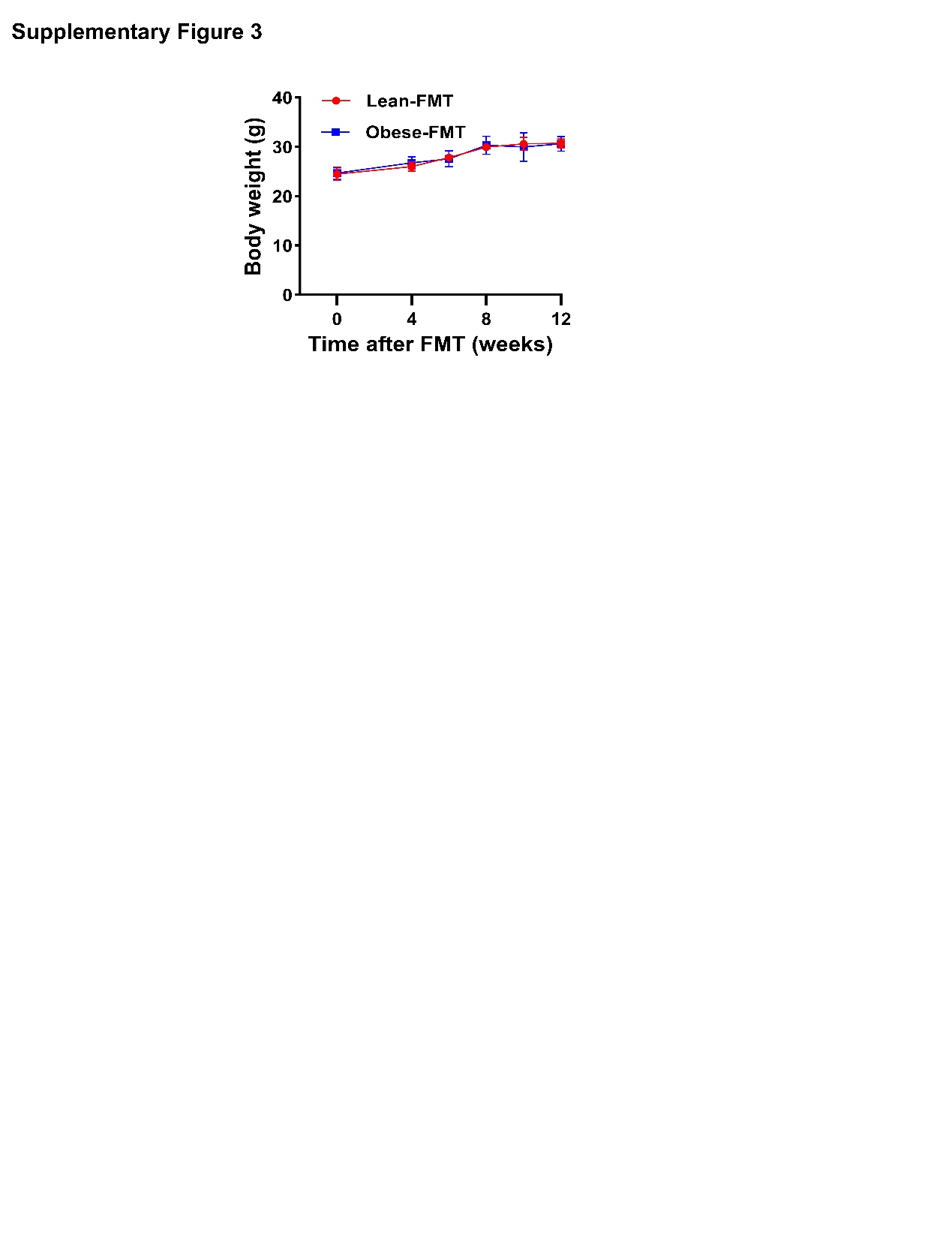
**

**Figure S3. Body weight of recipient mice with fecal microbial transplantation**

Line graph showing the changes of body weight (n = 6).

Data are shown as the mean ± SD. **P* <0.05; ***P* <0.01; ****P* <0.001; Student’s *t* test.

**
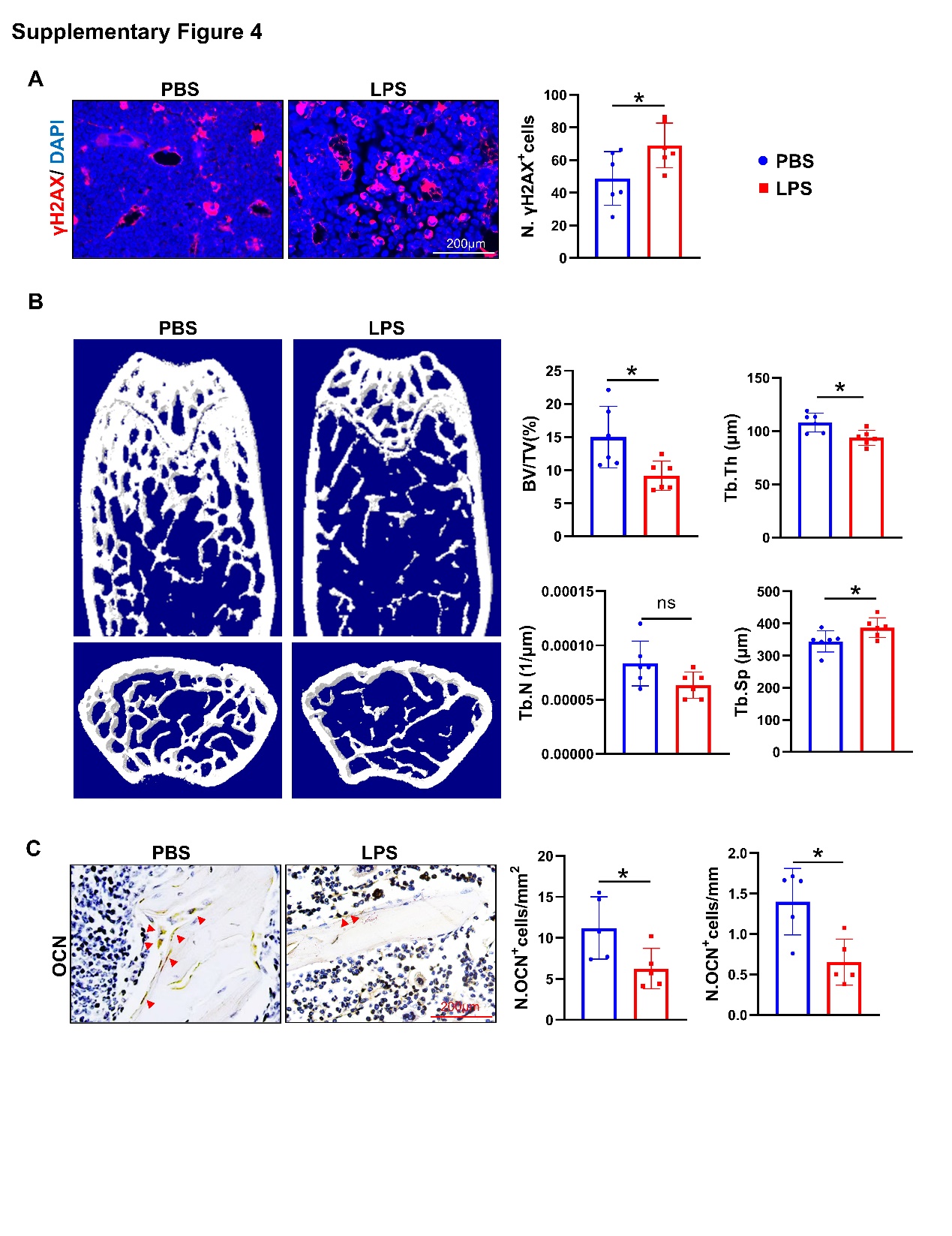
**

**Figure S4. LPS-induced chronic low inflammatory mouse model displays skeletal deterioration**

(A) 9 weeks old C57BL/6 mice were subjected to intraperitoneal injections of LPS (500 µg/kg, twice a week) or vehicle for a duration of 14 weeks. Representative immunofluorescence images of femur, immunostained with γH2AX (red) antibodies and counterstained with DAPI (blue) (scale bar = 200 μm, n = 6).

(B) Representative micro-CT images of femurs and quantitative analysis of BV/TV, Tb.Th, Tb.N and Tb.Sp (n = 6).

(C) Representative images of OCN staining and quantification of the number of osteoblasts (scale bar = 200 μm, n = 5).

Data are shown as the mean ± SD. **P* <0.05; ***P* <0.01; ****P* <0.001; Student’s *t* test.

**
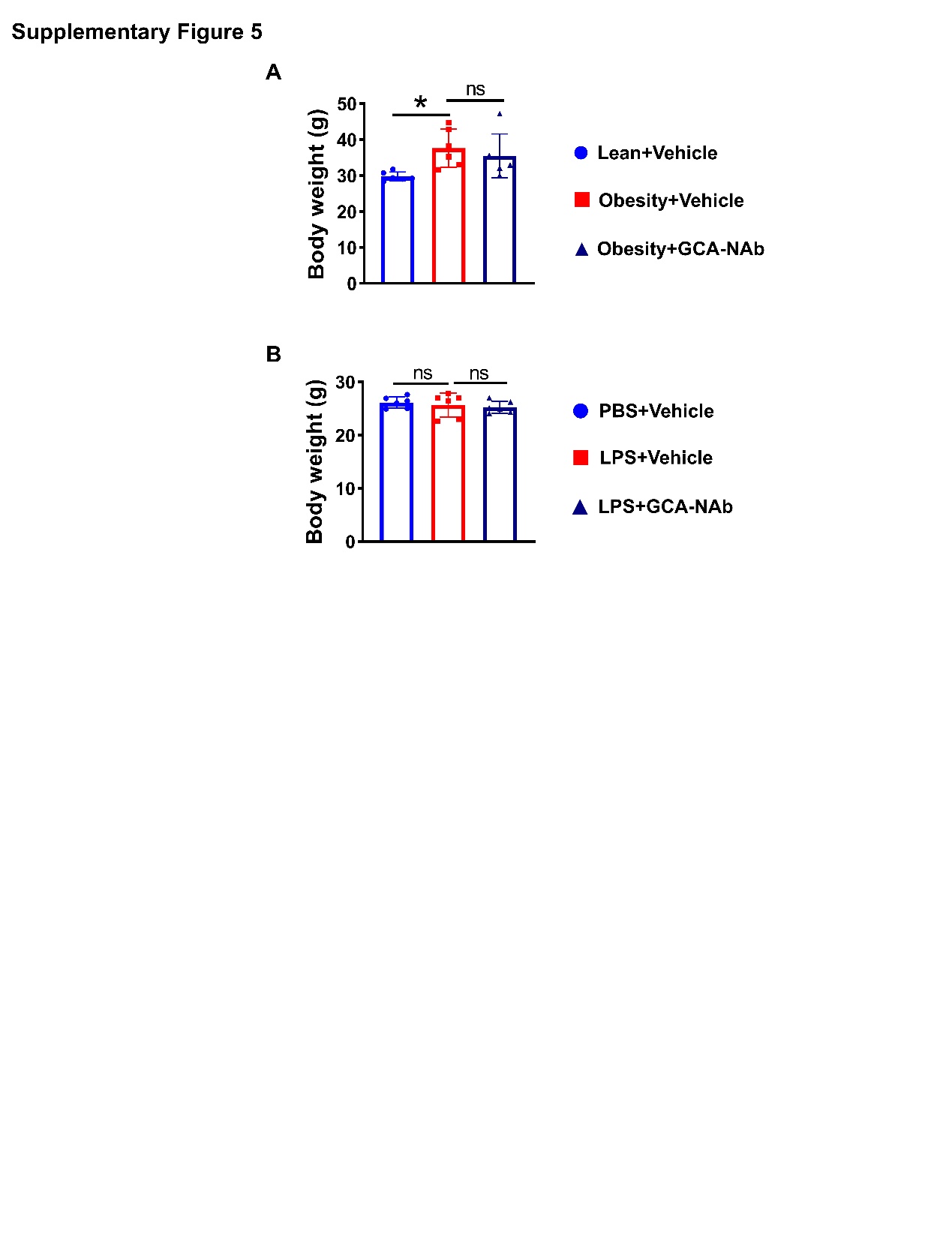
**

**Figure S5. The effects of GCA-neutralizing antibody on body weight**

GCA-Nab had no effect on the body weight of obese mice (A) or LPS-treated mice (B) (n = 6).

Data are shown as the mean ± SD. **P* <0.05; ***P* <0.01; ****P* <0.001; one-way ANOVA.

**Supplementary tables**

**Table S1. Characteristics of the Study Population**

| Trait | Individuals without obesity (n = 20) | Individuals with obesity (n = 20) | *P* value |
| --- | --- | --- | --- |
| Age (years) | 29.85±11.02 | 29.30±12.23 | 0.8820 |
| Male/female (n) | 8/12 | 8/12 | - |
| BMI (kg/m^2^) | 21.15±1.857 | 32.31±3.587 | ＜0.0001 |
| FBG (mmol/L) | 5.025±0.5957 | 5.265±0.6078 | 0.2158 |
| Triglycerides (mmol/L) | 1.105±0.3317 | 1.632±0.9562 | ＜0.05 |
| Cholesterol (mmol/L) | 3.752±0.8554 | 4.355±0.7259 | ＜0.05 |
| Creatinine (μmol/L) | 59.97±11.49 | 62.76±15.90 | 0.5284 |
| ALT (U/L) | 21.75±6.881 | 25.41±11.53 | 0.2310 |
| AST (U/L) | 21.65±4.405 | 22.70±7.492 | 0.5893 |

BMI: Body mass index; FBG: Fasting blood glucose;

ALT: Alanine aminotransferase; AST: Aspartate aminotransferase.

**Table S2. Primer sequences for qPCR**

| Gene Name (mouse) | Primers |
| --- | --- |
| *Cdkn1a* | Forward 5’-CTCGGCGTTTAATTTGGGAGA-3’ |
|  | Reverse 5’-TCGAGAGGGAGGTATTCTGAGT-3’ |
| *Cdkn2a* | Forward 5’-CAGTGTGACCGAAGATACCTGG-3’ |
|  | Reverse 5’-TCGAGACTTGATAGGGACCCC-3’ |
| *Trp53* | Forward 5’-CACTCCCACCCTGAGATTTGT-3’ |
|  | Reverse 5’-CATCGTCTGCACGGTTCTG-3’ |
| *Gca* | Forward 5’-GCGTCGTGATCCCCACTTAC-3’ |
|  | Reverse 5’-CAGGCCGAATAGGAGCGTC-3’ |
| *Alp* | Forward 5’-CCCCATGTGATGGCGTAT-3’ |
|  | Reverse 5’-CGGTAGGGAGAGCACAGC-3’ |
| *Runx2* | Forward 5’-GAAATGCCTCCGCTGTTATG-3’ |
|  | Reverse 5’-AGGTGAAACTCTTGCCTCGTC-3’ |
| *Sp7* | Forward 5’-ATGGCGTCCTCTCTGCTTG-3’ |
|  | Reverse 5’-TGAAAGGTCAGCGTATGGCTT-3’ |
| *Actin* | Forward 5’-GGCTGTATTCCCCTCCATCG-3’ |
|  | Reverse 5’-CCAGTTGGTAACAATGCCATGT-3’ |
| *Bglap* | Forward 5’-AAGCAGGAGGGCAATAAGGT-3’ |
|  | Reverse 5’-ATGCGTTTGTAGGCGGTCTT-3’ |
